# The Lectin LecA Sensitizes the Human Stretch-Activated Channel TREK-1 but Not Piezo1 and Binds Selectively to Cardiac Non-myocytes

**DOI:** 10.3389/fphys.2020.00457

**Published:** 2020-05-15

**Authors:** Elisa Darkow, Eva A. Rog-Zielinska, Josef Madl, Annette Brandel, Lina Siukstaite, Ramin Omidvar, Peter Kohl, Ursula Ravens, Winfried Römer, Rémi Peyronnet

**Affiliations:** ^1^Institute for Experimental Cardiovascular Medicine, University Heart Center Freiburg-Bad Krozingen, Medical Center-University of Freiburg, Freiburg, Germany; ^2^Faculty of Medicine, University of Freiburg, Freiburg, Germany; ^3^Spemann Graduate School of Biology and Medicine (SGBM), University of Freiburg, Freiburg, Germany; ^4^Faculty of Biology, University of Freiburg, Freiburg, Germany; ^5^Signalling Research Centres BIOSS and CIBSS, University of Freiburg, Freiburg, Germany

**Keywords:** mechano-sensitive channels, cardiomyocytes, mechanotransduction, membrane curvature, α-galactosides

## Abstract

The healthy heart adapts continuously to a complex set of dynamically changing mechanical conditions. The mechanical environment is altered by, and contributes to, multiple cardiac diseases. Mechanical stimuli are detected and transduced by cellular mechano-sensors, including stretch-activated ion channels (SAC). The precise role of SAC in the heart is unclear, in part because there are few SAC-specific pharmacological modulators. That said, most SAC can be activated by inducers of membrane curvature. The lectin LecA is a virulence factor of *Pseudomonas aeruginosa* and essential for *P. aeruginosa*-induced membrane curvature, resulting in formation of endocytic structures and bacterial cell invasion. We investigate whether LecA modulates SAC activity. TREK-1 and Piezo1 have been selected, as they are widely expressed in the body, including cardiac tissue, and they are “canonical representatives” for the potassium selective and the cation non-selective SAC families, respectively. Live cell confocal microscopy and electron tomographic imaging were used to follow binding dynamics of LecA, and to track changes in cell morphology and membrane topology in human embryonic kidney (HEK) cells and in giant unilamellar vesicles (GUV). HEK cells were further transfected with human TREK-1 or Piezo1 constructs, and ion channel activity was recorded using the patch-clamp technique. Finally, freshly isolated cardiac cells were used for studies into cell type dependency of LecA binding. LecA (500 nM) binds within seconds to the surface of HEK cells, with highest concentration at cell-cell contact sites. Local membrane invaginations are detected in the presence of LecA, both in the plasma membrane of cells (by 17 min of LecA exposure) as well as in GUV. In HEK cells, LecA sensitizes TREK-1, but not Piezo1, to voltage and mechanical stimulation. In freshly isolated cardiac cells, LecA binds to non-myocytes, but not to ventricular or atrial cardiomyocytes. This cell type specific lack of binding is observed across cardiomyocytes from mouse, rabbit, pig, and human. Our results suggest that LecA may serve as a pharmacological tool to study SAC in a cell type-preferential manner. This could aid tissue-based research into the roles of SAC in cardiac non-myocytes.

## Introduction

Cardiac tissue continuously experiences mechanical stimulation, and it must adjust structurally and functionally to an ever-changing mechanical environment. Key molecular players underlying mechano-sensing at the cellular level are stretch-activated ion channels (SAC). In the heart, activity and regulation of SAC are still poorly understood.

SAC are mechano-gated, i.e., their open probability can be increased by raising membrane tension (in-plane stretch) and/or curvature (Murthy et al., 2017). Bavi and co-workers, using a finite element model of cell membranes, concluded that local membrane curvature with a radius < 100 nm is required for SAC activation (Bavi et al., 2016). Under physiological conditions, membrane curvature in this order of magnitude is found at many different sites (e.g., in T-tubules, caveolae, filopodia), and in the context of various forms of cellular activity (e.g., endo- or exocytosis, cell division, migration). Under pathological conditions, membrane integrity and membrane protein functions can be adversely affected by changes in membrane curvature (e.g., Diabetes mellitus type 2, Alzheimer’s and Parkinson’s disease; Perlmutter et al., 2009; Kegulian et al., 2015; Sugiura et al., 2015).

SAC are conserved throughout living organisms and expressed in every human tissue tested so far, including the cardiovascular system (Peyronnet et al., 2016; Beech and Kalli, 2019; Douguet et al., 2019). SAC, both in the sarcolemma and as part of intracellular membrane systems, are believed to be indispensable molecular players in cardiac physiology, involved in the positive chronotropic response of the heart to stretch, auto-regulation of myocardial calcium balance and contractility, and vascular remodeling (Cooper et al., 2000; Youm et al., 2005; Iribe et al., 2009; Murthy et al., 2017). Perhaps even better documented is the involvement of SAC in pathological responses, contributing to the genesis of arrhythmias and fibrosis (Inoue et al., 2006; Peyronnet et al., 2016; Decher et al., 2017; Quinn et al., 2017; Abraham et al., 2018).

In mammals, two main families of SAC can be distinguished, based on their ion selectivity (Reed et al., 2014): cation non-selective channels (SAC_NS_) such as Piezo1, and potassium (K^+^) selective channels (SAC_K_) such as TREK-1 (TWIK-1-related K^+^ channel, where TWIK stands for “two-pore-domain weak inward rectifying K^+^ channel”). Piezo1 is involved in various cellular functions, including cell migration and alignment (Li et al., 2014), and it has been associated with human diseases such as hemolytic anemia, and lymphedema (Murthy et al., 2017). Piezo1 expression has recently been reported in the mammalian heart (Liang et al., 2017), more specifically in fibroblasts (Blythe et al., 2019), but its function and relevance are still unclear. TREK-1 is also widely expressed and present in the heart, both in non-myocytes and in myocytes (Terrenoire et al., 2001; Niu and Sachs, 2003; Abraham et al., 2018). A recent study showed that a mutation affecting TREK-1 permeability may lead to ventricular tachycardia (Decher et al., 2017). The function of “normal” TREK-1 in the heart is still unclear. Cardiac SAC have recently been reviewed in detail (Peyronnet et al., 2016), highlighting the need for new SAC-selective pharmacological modulators. Taking this thought a step further, cell type-specific SAC modulators would be of high utility in exploring cellular contributors to complex responses in heterocellular tissue.

A variety of pharmacological compounds have been identified as being able to modulate SAC. The vast majority of them are non-selective inhibitors (Hamill and McBride, 1996; Peyronnet et al., 2016). Among SAC-activators, membrane curvature inducers, such as the polyunsaturated fatty acid arachidonic acid (AA), are very efficient in shifting the stretch-dependence of SAC open probability to lower mechanical stimuli amplitudes. AA has been shown to influence ion channel activity in the cardiovascular system, including effects on TREK-1 in rat cardiomyocytes (Terrenoire et al., 2001; Li et al., 2006; Zhang et al., 2008). However, fatty acids (including AA) can modify the lipid composition of the bilayer, which in its own right may alter relevant membrane properties such as fluidity (Yang et al., 2011), or affect the lipid organization in ways that may generate side effects, such as changes in the activity of integral membrane proteins, cytotoxicity and apoptosis (Chen et al., 1998; Lee, 2004).

When employing lectin proteins to enhance membrane curvature, the lipidic composition of the bilayer is not altered. Lectins link *selectively* to glycoconjugates on the extracellular surface of various cell types. Some lectins bind specifically to glycosphingolipids with α-galactose residues, such as the glycosphingolipid globotriaosylceramide (Gb_3_). Gb_3_ has been reported to reside mainly in highly ordered lipid nanodomains, referred to as lipid rafts (Lingwood and Simons, 2010). Some lectins are able to change membrane curvature, as exemplified by the B-subunit of Shiga toxin, which has been identified as inducing membrane curvature by binding to Gb_3_ (Römer et al., 2007; Johannes and Römer, 2010; Johannes et al., 2015; Schubert and Römer, 2015; Sych et al., 2018).

The lectin LecA is a homo-tetrameric, brick-shaped lectin with four carbohydrate binding sites at two opposing ends (Schubert and Römer, 2015). LecA acts as a virulence factor of the Gram-negative bacterium *Pseudomonas aeruginosa*, an opportunistic pathogen involved in nosocomial infections which can also cause endocarditis. After binding to Gb_3_, the LecA-coated bacterium is able to induce concave (negative) membrane bending, which then triggers *P. aeruginosa* uptake into the host cell by endocytosis. Thus, LecA is not merely an adhesion factor, but it promotes the bacterial invasion process (Chemani et al., 2009; Aigal et al., 2015) by triggering the formation of membrane invaginations (Eierhoff et al., 2014). Since LecA binds selectively to membrane domains exhibiting α-galactose residues, the compound could be a candidate for modulating SAC in a cell type-selective manner.

From our current knowledge of SAC modulation, and of LecA, the following hypotheses are proposed: (i) LecA binds to human embryonic kidney (HEK) cells and induces membrane invaginations, (ii) LecA-induced membrane deformation affects SAC activity, and (iii) LecA binding is cell type-dependent.

## Materials and Methods

### Cell Culture

HEK 293T/17 cells (ATCC-LGC Standards, United States) were cultured in Dulbecco’s modified (low glucose) Eagle medium, supplemented with 10% fetal bovine serum and 1% penicillin/streptomycin (all Sigma Aldrich, Germany) in 25 cm^2^ tissue culture flasks (Techno Plastic Products, Switzerland) until 90% confluency. After washing with Dulbecco’s phosphate-buffered saline (PBS; Sigma Aldrich), cells were detached with trypsin (trypsin-EDTA solution; Sigma Aldrich). The suspension was centrifuged (57 × g, 3 min; Rotina 380, Hettich, Germany), the supernatant removed, and the pellet re-suspended in the cell culture medium described above. Cells were re-seeded for subculture or for experiments at 25,000 cells per Ø 35 mm dish (tissue culture or microscopy dishes; Techno Plastic Products or ibidi, Germany, respectively). Cells were cultured for a minimum of 2 days to allow cell attachment and expression of the protein of interest before further experiments were performed.

For transient overexpression of human Piezo1 or human TREK-1, HEK cells were transfected 24 h after seeding. Plasmids were kindly provided by Fred Sachs for Piezo1-internal ribosome entry site 2 (IRES2)-enhanced green fluorescent protein (eGFP), and by Eric Honoré and Delphine Bichet for TREK-1-IRES2-eGFP. For transfection of one dish, 0.5 μg of Piezo1-IRES2-eGFP or 0.1 μg of TREK-1-IRES2-eGFP were mixed with 1.5 μL of jetPEI transfection reagent in 100 μL NaCl (150 mM; both Polyplus Transfection, France). After incubating this transfection mix for 20 min at room temperature (RT), it was added to the cells (100 μL in 2 mL). Functional analyses were performed from days 2 to 4 post-transfection. Transfection success rate was about 50% (fluorescent cells per total number of cells).

### Lectins

Recombinant LecA production and purification were performed as described earlier (Blanchard et al., 2008) by transforming *Escherichia coli* BL21 (DE3) with the LecA-encoding plasmid pET25-pa1l. Cells were cultured in lysogeny broth medium (Carl Roth, Germany) with ampicillin (100 mg/mL) at 37°C under gentle agitation (for oxygenation; C25 KC, New Brunswick Scientific, United Kingdom) until the optical density of the cell suspension reached 0.8 at 600 nm (mid-logarithmic phase of cell growth). For induction of protein expression, isopropyl β-D-1-thiogalactopyranoside (1 mM) was added for 3 h at 30°C during continued gentle agitation. Then, cells were harvested and re-suspended in 10 mL loading/equilibration buffer (100 mM NaCl, 100 μM CaCl_2_, 20 mM Tris/HCl, pH 7.5; Carl Roth). For bacterial lysis, cells were disrupted in a high-pressure cell homogenizer at 170 MPa (CF1, Constant Systems, United Kingdom). After centrifugation (5,000 × g for 30 min at 4°C; Avanti J-26 XP, Beckman Coulter, Germany), the supernatant was passed through a 6 μm vacuum filter. The supernatant was further purified by affinity chromatography on D-Galactose columns (GE Healthcare Life Sciences, Germany). Lectins were allowed to bind to the immobilized saccharides, and were then eluted with elution buffer (100 mM NaCl, 100 mM EDTA, 20 mM TRIS/HCl, pH 7.5; all Carl Roth). The purity of the recombinant proteins was determined by gel electrophoresis (Monomeric LecA shows a band at its molecular weight of around 13 kDa; material from Bio-Rad, Germany). The purified proteins were intensively dialyzed against distilled water and CaCl_2_ (5 mM) for 3 days and against distilled water only for further 3 days. The bath solution was exchanged twice daily for sugar removal. The purified and lyophilized recombinant lectin was prepared in PBS (supplemented with 0.9 mM Ca^2+^ and 0.5 mM Mg^2+^; PBS^+/+^; Thermo Fisher Scientific) and aliquots were stored at −80°C.

The concentration at which lectins produce an effect may vary between artificial bilayer systems and cells, and it may also be different between various cell lines. On GUV, 200 nM of the B-subunit of Shiga toxin (StxB) induced tubular membrane invaginations (Römer et al., 2007). For LecA, 40-100 nM is sufficient to trigger a host cell signaling cascade (Eierhoff et al., 2014; Zheng et al., 2017). *P. aeruginosa*-induced lung injury was reproduced with 2 μM LecA (Chemani et al., 2009). Based on this, 500 nM was chosen as the target LecA concentration for HEK cells.

For imaging, labeling of purified LecA with Cyanine Dye 3 (Cy3; GE Healthcare Life Sciences) was performed according to the fluorophore manufacturer’s protocol. In short, fivefold molar excess of Cy3 was used. First, Cy3 was dissolved in dimethyl sulfoxide (anhydrous, Thermo Fisher Scientific) at 10 g/L. Then, the desired amount of LecA was mixed at 1:10 with 1 M NaHCO_3_, pH 8.7 (Carl Roth) and the calculated amount of Cy3, and the mixture was incubated for 1 h at RT with gentle agitation (Thriller Thermoshaker Incubator, Peqlab, Germany). To separate labeled LecA from non-bound Cy3, Spin Desalting Columns (Zeba, 7K MWCO, 0.5 mL, Thermo Fisher Scientific) were used. After washing with PBS (Thermo Fisher Scientific), the sample was centrifuged at 1,500 × g for 2 min at RT (Heraeus Fresco 21 Microcentrifuge, Thermo Fisher Scientific). After labeling, concentrations were determined by a spectrophotometer (NanoDrop 2000c, Thermo Fisher Scientific).

### Confocal Microscopy

An inverted confocal microscope (TCS SP8 X, Leica Microsystems, Germany) was used for live cell imaging. The 540 nm line of a white light laser was used to excite Cy3; in addition, a 405 nm diode laser was used when Hoechst33342 nucleus staining was assessed. The focal plane was set at the maximal cell cross-section area. Image acquisition and analysis were performed with the Leica Application Suite X software.

To follow dynamic changes in membrane configuration induced by LecA, HEK cells were imaged either in dishes with polymer coverslip bottom (μdish Ø 35 mm, ibidi) or on round glass coverslips (Ø 30 mm, thickness N° 1.5, Bioptechs) mounted in an interchangeable coverslip dish (Bioptechs, United States). In order to reduce the volume of the measurement chamber, clean, 10 mm-high Teflon tube pieces were glued onto these coverslips with a two-component silicone adhesive (Reprorubber, United States; Madl et al., 2016). These home-built reduced volume chambers (∼200 μL instead of 1 mL) were disinfected using 70% ethanol before seeding cells.

Live cell imaging was performed 3 d after seeding of cells. Labeled LecA (500 nM, in PBS^+/+^) was added to HEK cells at RT and imaged within the first minute [average delay 30 s, 63× oil objective, numerical aperture (NA) 1.4]. For time-lapse microscopy, one focal plane was imaged every 10 s. Fluorescence analyses were performed with ImageJ software. Cell maximum cross sectional area and mean fluorescence were determined. Fluorescence per cell is defined as the product of cell cross sectional area and mean fluorescence (of a cell cluster divided by the area of the cluster), and assumed to be proportional to the total amount of protein in the selected area. Cell selection criteria were: clearly defined borders in at least one channel, and cell attachment to the dish (which correlated with the presence of membrane protrusions). Only cells that could be observed throughout the entire time-lapse protocol were included in the analysis.

To determine cell type dependency of LecA binding, isolated cardiac cells were imaged in small chambered coverglass wells (μ-Slide 8 Well, ibidi). After Hoechst33342 staining of nuclei (20 μM, 7 min, in PBS^+/+^; Thermo Fisher Scientific), cells were incubated with LecA (500 nM, in PBS^+/+^) for 1 h at RT to maximize potential binding and avoid false negatives. To get an overview, a 40 × water objective or a dry 10 × objective were used. Only cells that appeared viable, i.e., showing a sharp and contrasted plasma membrane/sarcolemma, a well-delineated nucleus, and – in case of cardiomyocytes – clear non-contracted cross-striations (sarcomere length ≥ 1.7 μm), were considered.

### Electron Microscopy, Tomographic Imaging, and Correlative Light-Electron Microscopy

HEK cells were grown on 6 mm sapphire discs (thickness 100 μm), and preserved by chemical fixation using iso-osmotic Karnovsky’s fixative (Karnovsky, 1965; Solmedia, United Kingdom). Samples were processed to Epon-Araldite resin as described before (Rog-Zielinska et al., 2018). Imaging was performed at the Electron Microscopy (EM) Core Facility at the European Molecular Biology Laboratory in Heidelberg, using a 300 kV Tecnai TF30 microscope (FEI Company, now Thermo Fisher Scientific, Netherlands) and a 4k × 4k charge-coupled device camera (Oneview, Gatan, Germany) for dual and single-tilt 3D electron tomography (ET) of thick (300 nm) sections, or a 120 kV Biotwin CM120 microscope (Philips, Thermo Fisher Scientific) equipped with a bottom-mounted 1K CCD Camera (Keen View, SIS, Olympus, Germany) for 2D transmission EM of thin (70 nm) sections. Data were visualized and analyzed using IMOD routines, as described before (Kremer et al., 1996; Rog-Zielinska et al., 2018).

For correlative light-electron microscopy (CLEM), we used HEK293 cells, cultured on carbon- and fibronectin-coated sapphire discs (thickness 160 μm). Cells were treated with LecA for 17 min. Sapphire discs were high-pressure frozen (EM ICE, Leica Microsystems, Austria), and freeze-substituted in 0.1% uranyl acetate in acetone and embedded in Lowicryl HM20 resin (Polysciences, Germany) as described before (Santarella-Mellwig et al., 2018). Thick sections (300 nm thick) were cut, placed on carbon-coated copper mesh grids, and fluorescence-imaged using a widefield fluorescence microscope (IX81, 100× oil immersion objective, NA 1.4, Olympus) and a CCD camera (Orca-ER, Hamamatsu Photonics, Germany). Sections were then post-stained with uranyl acetate and lead citrate. ET was performed on the Tecnai TF30 microscope as described above. Fine registration was conducted using the ec-CLEM function of the Icy software (De Chaumont et al., 2012; Paul-Gilloteaux et al., 2017). As a note of caution, care must be taken when interpreting CLEM images. CLEM inherently involves the matching of datasets with vastly different resolutions (ET voxel size is ∼10^0^ nm^3^, whereas for light microscopy a diffraction-limited voxel size is >10^6^ nm^3^). This poses challenges in terms of different granularity of information, addressed post-acquisition using advanced image processing tools to achieve the correlation and cross-mapping of the two datasets. This, in turn, can generate an inaccurate impression of the information content in the fluorescence channel (e.g., appearing able to generate signals at a resolution well below the diffraction limit of light).

### GUV Preparation and Observation

GUV were composed of 1,2-dioleoyl-sn-glycero-3-phosphocholine (DOPC), cholesterol (both Avanti Polar Lipids, United States), Atto 647N 1,2-dioleoyl-sn-glycero-3-phosphoethanolamine (DOPE; Sigma Aldrich), and purified porcine Gb_3_ (Matreya, United States) at a molar ratio of 64.7: 30: 0.3: 5 mol%.

GUV were prepared using the electroformation technique as previously described (Madl et al., 2016). Briefly, lipids dissolved in chloroform were deposited on indium tin oxide (ITO)-coated glass slides (Präzisions Glas and Optik, Germany). After complete evaporation of chloroform solvent, lipids were hydrated with 300 mM sucrose in a pre-assembled chamber. GUV were formed by applying an alternating voltage (Vmax = 1.1 V) to the chamber for 2-3 h.

GUV were imaged on an inverted confocal microscope (Eclipse Ti-E, Nikon, Japan; with a Nikon A1R confocal laser scanning system, and 60× oil immersion objective, NA = 1.49). The software NIS-elements (Nikon) was used for image acquisition and ImageJ for analysis.

### Patch-Clamp

Electrophysiological properties of SAC in the plasma membrane of HEK cells, and their response to drug application, were investigated using the patch-clamp technique in voltage clamp mode at RT. Cells were exchanged every hour.

The dish containing cells was mounted onto an inverted microscope (40× objective; DM IRB, Leica Microsystems). Transfected cells were identified by cytosolic eGFP fluorescence upon illumination with a mercury lamp (HBO100, Leica Microsystems). For measurements, cells with moderate and even fluorescence intensities were chosen. Fire-polished soda-lime glass capillaries (inner diameter: 1.15 ± 0.05 mm, outer diameter: 1.55 ± 0.05 mm; VITREX Medical, Denmark) were pulled using a two-stage vertical pipette-puller (PC-10, Narishige, Japan) to create the micropipettes required for patch-clamping. Pipette resistance depended on application (see below).

Pressure in the pipette line was recorded with a pressure monitor (PM01D, World Precision Instruments, United States). Cells were approached with positive pipette pressure (5–10 mmHg). To create a seal between the cell and the pipette upon cell contact, negative pressure (suction) was applied. Recordings were obtained in cell-attached or whole-cell configurations. A patch-clamp amplifier (Axopatch 200B, Axon Instruments, United States) and a digitizer interface (Axon Digidata 1440A, Axon Instruments) were used. Currents were acquired at 20 kHz sampling rate, and low-pass filtered at 1 kHz. Recordings were analyzed with pCLAMP 10.6 software (Axon Instruments).

For cell-attached configuration, bath solution contained (in mM): 155 KCl, 3 MgCl_2_, 5 EGTA, 10 N-2-hydroxyethylpiperazine-N-ethanesulfonic acid (HEPES); pH buffered to 7.2 using KOH, osmolarity ∼300 mOsm L^–1^ (K-7400, Knauer, Germany), to match intracellular ionic conditions. The solution was stored at RT. Pipette solution, formulated to mimic the extracellular ionic environment, contained (in mM): 150 NaCl, 5 KCl, 10 HEPES, 2 CaCl_2_, pH buffered to 7.4 using NaOH, ∼300 mOsm L^–1^. Pipette solution was prepared ahead of experiments and stored in aliquots at −20°C. These ionic conditions are standard to record Piezo and TREK-1 channels (Moha ou Maati et al., 2011; Peyronnet et al., 2012, 2013). In case of application of compounds, these were added to the pipette solution before use. A delay of 2 min after seal formation was allowed for LecA effects to develop.

To test for SAC_NS_ (reversal potential ≈0 mV), voltage was clamped to −80 mV. To test for SAC_K_ (reversal potential ≈−80 mV), voltage was clamped to 0 mV. Average pipette resistance for cell-attached recordings was 1.36 ± 0.03 MΩ (*n* = 164). Seal resistances accepted were ∼1 GΩ, independent of pipette size and cell quality. Local membrane stretch was induced by applying pulses of negative pressure through the patch pipette, generated by an automated precision system (High Speed Pressure Clamp system; HSPC-1, ALA Scientific Instruments, United States). For mechanical stimulation, negative pressure was applied for 500 ms, in 5 mmHg increments from 0 to −80 mmHg (i.e., 16 sweeps), with 1.8 s at pressure = 0 mmHg between pressure steps.

Recordings obtained with the stretch protocol were analyzed in Clampfit 10.6 (Molecular Devices, United States). Baseline activity at 0 mmHg was taken as the “zero” reference (average baseline current = 0.3 ± 1.57 pA; *n* = 259). Peak, average and near steady-state currents were analyzed during pressure pulses. The time constant (τ) was determined according to the exponential function $f\left(t\right)=\sum_{i=1}^{n}A_{i}ie^{\frac{-t}{\tau_{i}}}+c$, where A: amplitude, τ: time constant and c: y-offset. The time constant τ (in ms) reflects the speed of inactivation after initial activation of SAC (analysis parameters illustrated in Supplementary Figure S2A). Averaged data were fitted by the Boltzmann equation: $y=\frac{A_{1}-A_{2}}{1+e^{\left(x-x_{0}\right)/\tau }}+A_{2}$ (with A_1_: maximum current, A_2_: current near steady-state, x_0_: inflection point, τ: time constant).

For whole-cell configuration, solutions were as above, but inverted (pipette solution in cell-attached configuration constitutes the bath solution, and *vice versa*). Thereby, the pipette solution mimics the intracellular ionic milieu while the bath solution mimics extracellular ionic conditions.

A local perfusion system was installed for controlled administration of control (bath solution) or AA-containing solution (10 μM in bath solution; Meves, 2008). Flow rate was adapted by adjusting the height of reservoirs for gravity-fed flow to 1 mL min^–1^, yielding nominally one exchange of the bath per minute. Due to the limited availability of LecA, it was added manually to the bath solution at a final concentration of 500 nM (to avoid the dead space of the perfusion system).

Average pipette resistance for whole-cell studies was 1.97 ± 0.02 MΩ; *n* = 124. A voltage ramp protocol (continuous change from −120 V to +40 mV within 1 s) was used for electrical stimulation (potential gradients above +40 mV were found to compromise membranes). Between application of ramps, cells were held for 5 s at −80 mV. On average, 10 min of stable recordings were obtained per cell.

To compare recordings from different cells, currents obtained in whole-cell configuration were normalized to membrane capacitance (a surrogate for cell size) and expressed as current density (in pA/pF). Currents at the maximum level of de- and hyperpolarization (+40 and −80 mV) were analyzed for each ramp acquired.

### Cardiac Cell Isolation

For cell isolation, hearts were excised from animals sacrificed according to the guidelines stated in Directive 2010/63/EU of the European Parliament on the protection of animals used for scientific purposes, with approval by the local authorities in Baden Württemberg (G-16-131). Mouse and rabbit hearts were mounted on a Langendorff apparatus and perfused with collagenase-containing isolation solution (for protocols see Burton et al., 2017; Peyronnet et al., 2017). Porcine atrial and ventricular tissue was obtained with approval from the local ethics committee and the regional council of Freiburg, Baden-Württemberg, Germany (license number 35-9185.81/G-19/70). Human atrial tissue samples were obtained from patients in sinus rhythm, undergoing open heart surgery at the University Heart Center Freiburg-Bad Krozingen. All tissue is used with written informed consent and handled *via* the CardioVascular BioBank (CVBB) Freiburg, as approved by the Ethics Commission of the University of Freiburg, Freiburg, Germany (CVBB ethical approval reference 393/16 and 214/18). Human and porcine tissues were provided *via* the CardioVascular BioBank (CVBB study approval reference 214/18). Enzymatic cell isolation followed the protocol of Schmidt et al. (2017). Myocardial tissue was placed into Ca^2+^-free “Kraftbrühe” (Isenberg and Klockner, 1982); containing (in mM): 20 KCl, 10 KH_2_PO_4_, 25 glucose, 40 D-mannitol, 0.1% (w/v) albumin, 70 K-glutamate, 10 β-hydroxybutyrate, 20 taurine, 10 ethylene glycol tetraacetic acid (EGTA); pH adjusted to 7.4 using KOH, ∼300 mOsm L^–1^. Tissue was cut into pieces of 1 mm^3^, and rinsed for 5 min with pre-digestion solution (in mM): 137 NaCl, 5 KH_2_PO_4_, 1 MgSO_4_ (7H_2_O), 10 glucose, 10 taurine, 5 HEPES, 0.2 EGTA; pH adjusted to 7.4 using NaOH, ∼300 mOsm L^–1^). Solutions were oxygenated with 100% O_2_ at 37°C and stirred. For digestion, tissue pieces were transferred for 10 min into digestion solution [as pre-digestion solution, but without EGTA, and supplemented with albumin (0.1%), collagenase type V (200 U mL^–1^) and proteinase type XXIV (5.4 U mL^–1^); all Sigma Aldrich]. After induction of enzyme activity by increasing the Ca^2+^ concentration to 0.02 mM, tissue chunks were agitated for further 20–30 min and then transferred into protease-free cell isolation solution (containing collagenase and 0.02 mM Ca^2+^) for another 10 min. Dissociation of single cells was checked under a light microscope. The last dissociation step was repeated until cardiomyocytes with clear cross striations appeared. Digestion was stopped by removing the supernatant when 2–3 cross-striated cells per field of view were observed at high magnification (40× objective). Cell suspensions were centrifuged (2 min at 7 × g; PK121R, ALC, United Kingdom), and myocytes were re-suspended in Kraftbrühe without EGTA at RT, until use. If enrichment in non-myocytes was needed, centrifugation at 260 × g for 5 min was performed, to spin down cardiomyocytes and to enrich the supernatant in non-myocytes.

### Statistics

If not specified otherwise, data are presented as mean ± standard error of the mean. For statistical analysis, Origin 2019 software (OriginLab Corporation, United States) was used; graphic representations were made using Origin and Canvas X software (ACD Systems, United States). “N” indicates the number of independent experiments (e.g., the number of animals or tissue donors); “n” is the number of cells studied. In general, control data are shown in black, interventions in red.

Significance of the difference between two independent and normally distributed groups was tested with a two-tailed Student’s *t*-test if n ≥ 20, or with the non-parametric Mann–Whitney-test if n < 20. Prior to *t*-tests, each sample was tested for variance. If paired experiments were conducted (whole-cell configuration), means were compared using the paired *t*-test. To compare the presence or absence of membrane rupture, the Chi-square test was used if *n* ≥ 20, and Fisher’s exact test if *n* < 20. One asterisk indicates a *p-*value *p* ≤ 0.05 and this was considered indicative of a statistically significant difference between means; two asterisks: *p* < 0.01; three asterisks: *p* < 0.001; n.s. indicates that no significant differences are seen (*p* > 0.05).

Statistical power analysis was performed with GPower 3.1 software to identify the necessary sample size.

## Results

### LecA Binding Dynamics and Effects on Membrane Topology

**FIGURE 1:**
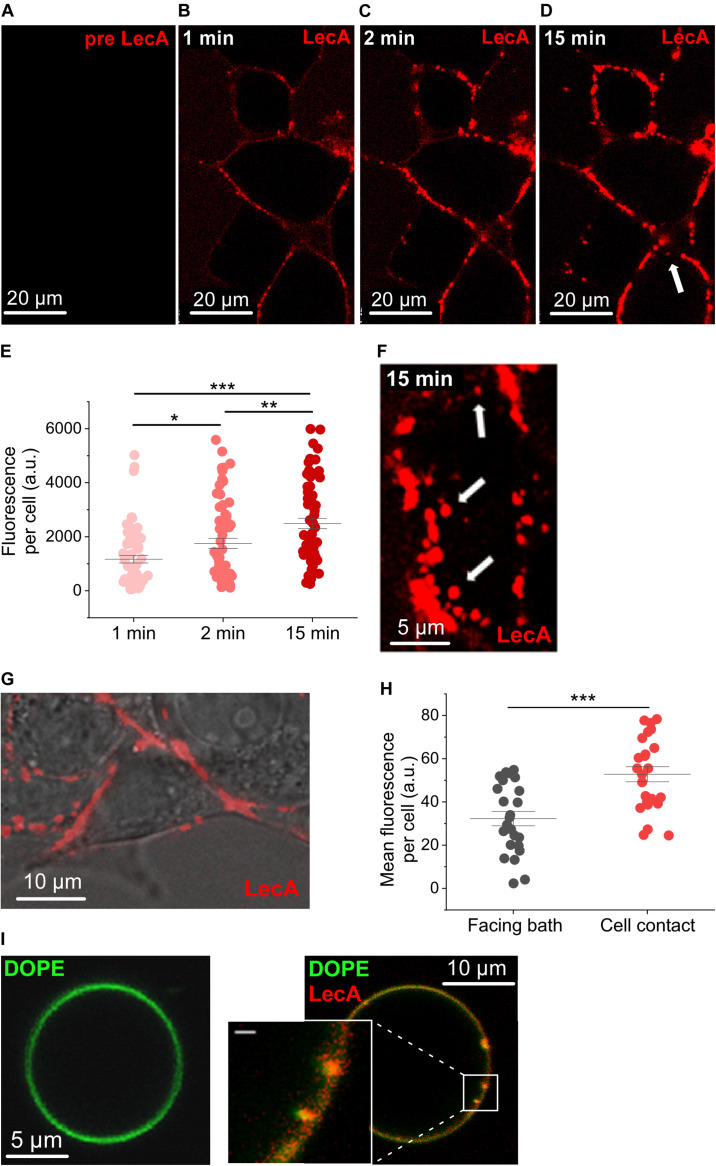
LecA binding to HEK cells and giant unilamellar vesicles. Representative confocal microscopy images (one plane) of HEK cells incubated with LecA (in red). **(A)** Before application of 500 nM LecA. **(B)** LecA within the first minute after application. **(C)** LecA accumulation at 2 min. **(D,F)** appearance of first endosomes at 15 min (arrows). **(E)** Quantification of the fluorescence of LecA per cell at 1, 2, and 15 min (*n* = 63; *N* = 6). Significance was assessed by the *t*-test. **(G)** Overlay of transmission light and fluorescence microscopy, illustrating LecA accumulation at cell-cell contact site and less LecA staining at the cell membrane facing the bath. **(H)** Quantification of the mean fluorescence of LecA per cell at membranes facing the bath or facing a neighboring cell (cell contact; *n* = 24; *N* = 6). Significance was assessed by the *t*-test. **(I)** Gb3-decorated giant unilamellar vesicle (Atto 647N 1,2-dioleoyl-sn-glycero-3-phosphoethanolamine (DOPE), green) without (left) and with (right) 500 nM of LecA (red). LecA (500 nM) binds to Gb_3_ and induces tubular membrane invaginations (inset, scale bar = 1 μm). *p ≤ 0.05, **p < 0.01, and ***p < 0.001.

Cy3-labeled LecA started to binds at the surface of HEK cells by the first time-point we could image after addition to the bath solution (Figures 1A–D), i.e., within less than 30 s (hereafter referred to as the first 1 min-bin). LecA binding was quantified as fluorescence per cell (a.u.) and amounted to 1,169.0 ± 140.3 at 1 min, rising to 1,753.6 ± 188.86 at 2 min (*n* = 63; *N* = 6; *p* = 0.014; Figure 1E). At 15 min, LecA intensity was 2,487.8 ± 190.4 (*n* = 63; *N* = 6; *p* = 0.007 compared to 2 min). At this time-point, the first internalized LecA vesicles were detected, and tubular invaginations or single labeled endosomes started to appear (arrows in Figures 1D,F). LecA accumulated preferentially at cell-cell contact sites compared to patches of membrane facing the bath solution (Figures 1G,H). Accumulation of LecA on the surface of HEK cells was clustered after 1 min of incubation (Figures 1B–D,F). Similar uneven distribution was also observed on GUV after the initial binding phase where LecA also induced concave membrane deformations (Figure 1I).

**FIGURE 2:**
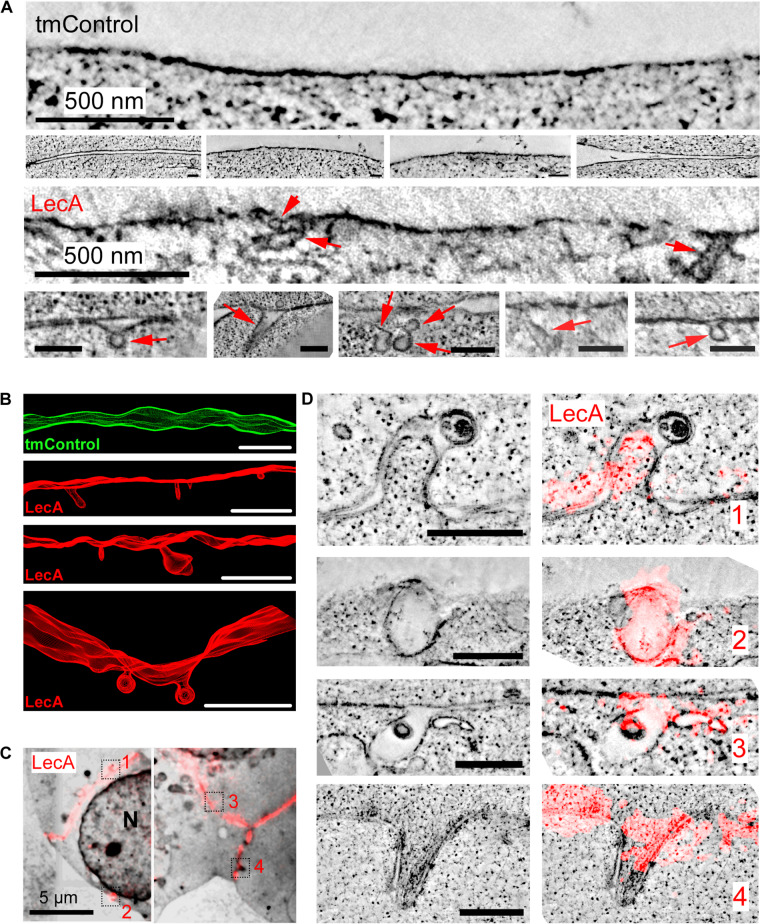
LecA-associated changes in membrane ultrastructure in HEK cells. **(A)** Representative 2D electron tomographic slices (maximum projection over 50 nm) of tmControl cells (top rows) and cells exposed for 17 min to 500 nM LecA (bottom rows), demonstrating the morphology of LecA-associated surface membrane invaginations. If not otherwise stated, scale bars = 200 nm. **(B)** Representative 3D electron tomographic reconstructions of surface plasma membrane in tmControl (top, green) and LecA-exposed cells (red), demonstrating numerous membrane invaginations. Reconstructed volume: (4,190 × 4,190 × 300) nm^3^; scale bars = 500 nm. **(C)** CLEM overview maps of LecA-exposed cells, demonstrating the preferential localization of the fluorescent signal at the cell-cell interfaces. The median free gap width between adjacent cells in areas of high fluorescence signal is 8.8 nm. Squares highlight the location of larger resolution images in **(D)**. N, nucleus. **(D)** Representative 2D electron tomographic slices (left) and corresponding CLEM images (right) of LecA-exposed cells. The slices demonstrate the colocalization of LecA staining (red) with plasma membrane invaginations on neighboring and single (second panel from above) cells. Scale bar = 500 nm.

EM and ET of cells chemically fixed after 17 min of LecA exposure revealed concave membrane invaginations; compatible in their shape with caveolae-like structures, while others are more tubular. These invaginations are not seen in time-matched controls (tmControl) in the absence of LecA (Figures 2A,B).

CLEM further demonstrated (i) the preferential localization of the fluorescent signal at cell-cell interfaces (Figure 2C), and the association of membrane invaginations with LecA fluorescence (Figure 2D). Membrane invaginations at cell-cell interfaces often presented as paired in/ex-vagination events of the two apposing cell membranes. While all membrane invaginations were associated with LecA fluorescence, LecA signal was also seen in membranes containing no curved domains, possibly owing to the fact that membrane curvature induction by LecA is a non-synchronized process that takes place over time.

### LecA Effect on SAC

In patch-clamp experiments, HEK cells transfected with Piezo1 or TREK-1 exhibited significantly higher SAC activity than non-transfected tmControl cells (for Piezo1 at all suction levels more negative than −10 mmHg; for TREK-1 even at rest; Supplementary Figure S1), indicating effective functional expression of the channels. Moreover, channel kinetics were similar to those published previously (Patel et al., 1998; Coste et al., 2010).

**FIGURE 3:**
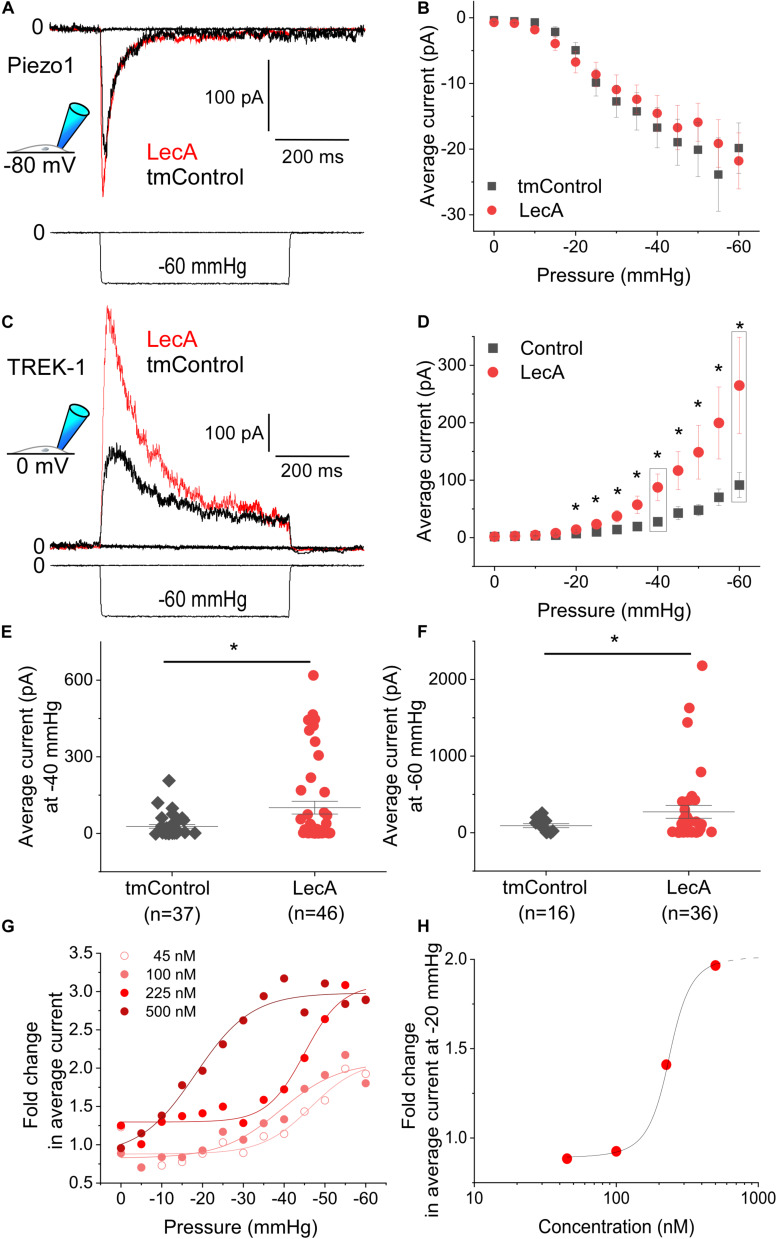
Mechanically-induced Piezo1 and TREK-1 activity in absence or presence of LecA in HEK cells. Patch-clamp measurements in cell-attached configuration showing Piezo1 **(A,B)** and TREK-1 **(C–H)** activity, at holding potentials of −80 and 0 mV, respectively, after 2 min of exposure to 500 nM LecA (red), compared to time-matched controls (tmControl, black). **(A)** Representative Piezo1 recording during application of negative pressure (mechanical activation) of −35 mmHg. **(B)** Quantification of Piezo1 activity in the absence or presence of LecA at 0 mmHg (*n* = 38 and 38, respectively) and −60 mmHg (*n* = 23 and 23, respectively, lower n-numbers in tmControl cells are caused by patches that did not withstand −60 mmHg suction levels). Significance assessed by the *t*-test. **(C)** Representative TREK-1 recording during application of negative pressure (mechanical activation) of −60 mmHg. **(D)** Quantification of TREK-1 activity in the absence and presence of LecA at 0 mmHg (*n* = 43 and 46, respectively) and −60 mmHg (*n* = 16 and 36, respectively; lower n-numbers in tmControl as above). Significance assessed by the *t*-test. **(E,F)** Single data points and means ± SEM of TREK-1 at −40 and −60 mmHg. Significance was assessed by the *t*-test. **(G)** Fold change (LecA/tmControl) in TREK-1 average current at suction levels from 0 to −60 mmHg for different LecA concentrations (45, 100, 225, and 500 nM). The averaged data were fitted with a Boltzmann function. **(H)** Concentration-response curve, the stretch-induced fold change (LecA/tmControl) in TREK-1 average current at −20 mmHg is presented as a function of LecA concentration. *p ≤ 0.05.

Piezo1 activity was not affected by LecA, neither in cell-attached, nor in whole-cell configurations. As an example: at a holding potential of −80 mV, the average current at −35 mmHg was −14.3 ± 2.8 pA in tmControl (*n* = 37) and −12.4 ± 2.2 pA in LecA-exposed cells (*n* = 37, *p* = 0.574; Figure 3A). Current-pressure relationships were not different in the two conditions (Figure 3B). The same held true for the time-constants of inactivation, peak current and near steady-state current (Supplementary Figures S2B–D). At a holding potential of +40 mV, current-pressure relationships were also not different between both groups (*n* = 23 each for LecA and the tmControl group, data not shown). The voltage ramp protocols applied in whole-cell configuration upon LecA exposure did not reveal any changes in Piezo1 current either. The ratio of current after LecA application to pre-drug control did not change from 0 to 80 s. Analyzed at +40 mV of the ramp pulse and after 80 s of LecA exposure, ratios were 1.04 ± 0.02 with LecA (*n* = 7) and 1.00 ± 0.01 in tmControl (*n* = 7, *p* = 0.371; Supplementary Figures S2E,F). At −80 mV, there was also no difference between both groups (data not shown).

In contrast to Piezo1, TREK-1 was sensitized to mechanical stimulation by LecA. In cell-attached configuration, this was significant for pressures more negative than −20 mmHg (Figures 3C,D). The average current at −40 mmHg was 27.6 ± 7.0 pA in tmControl (*n* = 37) and 87.6 ± 23.3 pA in cells exposed to 500 nM LecA for 2 min (*n* = 46, *p* = 0.007). Individual average currents measured at −40 and −60 mmHg are depicted in Figures 3E,F. Current-pressure curves for peak and near steady-state current were also significantly different with and without LecA, while the time constants of peak current decay were not (Supplementary Figure S3). At a holding potential of −120 mV, the current-pressure relationships were not different between both groups (*n* = 23 for the tmControl group and *n* = 23 cells in presence of LecA; data not shown).

**FIGURE 4:**
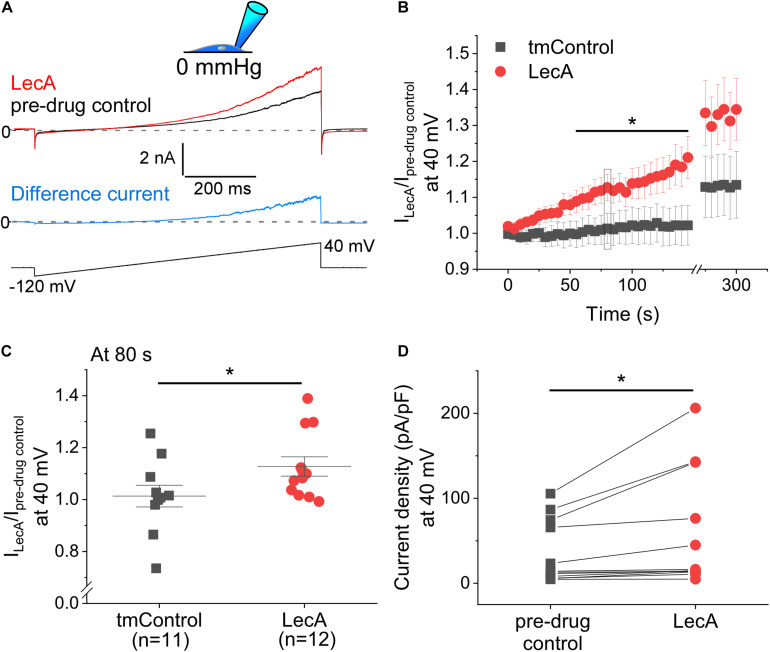
Voltage-induced TREK-1 activity in absence or presence of LecA in HEK cells. Patch-clamp measurements in whole-cell configuration (pipette pressure 0 mmHg). **(A)** Representative recording; Top: LecA at 80 s after onset of LecA exposure; pre-drug control 10 s before LecA exposure; Middle: Difference current (blue); pre-drug control activity subtracted from LecA activity; Bottom: voltage ramp applied from −80 to +40 mV. **(B)** Quantification of the activity induced by LecA vs. tmControl (*n* = 12 at 0 and 80 s in both groups). **(C)** Single data points (tmControl vs. LecA) at 80 s are shown. Significance was assessed by the Mann–Whitney-test. **(D)** Single paired data points (pre-drug control vs. LecA). The maximum density of LecA-induced current is shown. Significance was assessed by the paired *t*-test. *p ≤ 0.05.

The concentration-dependence of LecA effects on TREK-1 was tested in cell-attached configuration at a holding potential of 0 mV. While 45 and 100 nM of LecA had no effect after 2 min, 225 and 500 nM increased the TREK-1 response to stretch. As for the fold change (LecA/tmControl) in average current, the half maximal effective pressure was −46.6 mmHg at 45 nM, −39.1 mmHg at 100 nM, −45.0 mmHg at 255 nM, and −18.3 mmHg at 500 nM of LecA with *n* = 13, 50, 25, 46, respectively (a total of *n* = 133 tmControl recordings were conducted; Figure 3G). At −20 mmHg, the concentration-response curve is shown Figure 3H. In whole-cell configuration, the effect of LecA on TREK-1 during the first 80 s of exposure was determined with voltage ramp pulses. The LecA-induced current showed outward rectification (Figure 4A). The activity of TREK-1, relative to pre-drug control, increases with exposure time to LecA, with significant difference for all time points exceeding 55 s (Figure 4B). As an example, after 80 s and +40 mV of the ramp pulse, corresponding values were 1.13 ± 0.04 with LecA (*n* = 12) and 1.01 ± 0.04 in tmControl (*n* = 11, *p* = 0.039; Figure 4C). The propensity toward increasing TREK-1 current was visible on a cell-by-cell basis (*p* = 0.031) (Figure 4D). At −80 mV, there was no difference between both groups (data not shown).

Since the known membrane curvature inducer AA had been shown previously to reversibly activate TREK-1 (Fink et al., 1998), we also tested its effect. As expected, 10 μM of AA produced robust activation of TREK-1 in whole-cell recordings, which reversed slowly upon wash-out (Supplementary Figure S4). Interestingly, AA, like LecA, did not alter Piezo1 activity (Supplementary Figure S5).

### LecA Effect on Patch Stability

**FIGURE 5:**
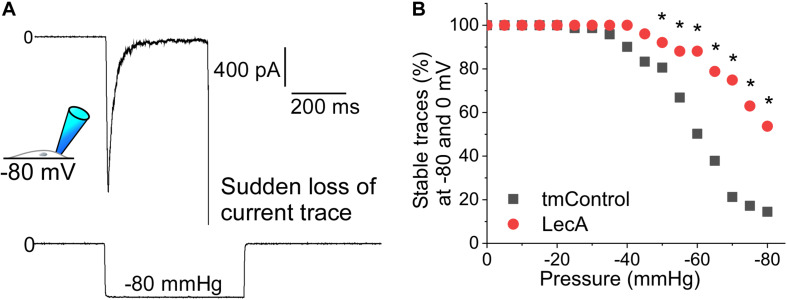
Patch stability in response to negative pressure in the absence or presence of LecA in HEK cells. Patch-clamp measurements in cell-attached configuration (data pooled from Piezo1 and TREK-1 recordings) after 2 min of LecA exposure. **(A)** Exemplary recording of a sudden loss of the current trace at −80 mmHg suction; here voltage clamped to −80 mV. **(B)** Quantification of the maximum pressure levels that were sustained in tmControl (*n* = 61 at 0 mmHg; *n* = 11 at −80 mmHg) vs. LecA (*n* = 71 at 0 mmHg; *n* = 38 at −80 mmHg). Significance was assessed by the chi-square test. *p ≤ 0.05.

Suction pulses of high negative pressure can destabilize a membrane patch and cause sudden loss of the current trace. We investigated the influence of LecA on the maximum negative pressure that patched cell membranes can withstand (Piezo1 and TREK-1 cell-attached recordings, pooled). In tmControl conditions, sudden loss of recordings started to occur at −35 mmHg. In the presence of LecA, higher negative pressure values were tolerated, so that first losses were observed at −45 mmHg. At −80 mmHg, only 14.5% of tmControl patches (*n* = 61) remained intact, whereas 53.7% of patches in the presence of LecA were sustained (*n* = 71; Figure 5). This difference was statistically significant for all pressure levels more negative than −45 mmHg (*p* = 0.021; chi-square test). This difference was maintained for in- and outward currents for both channels (data not shown).

### Cell Type Dependency of LecA Binding

**FIGURE 6:**
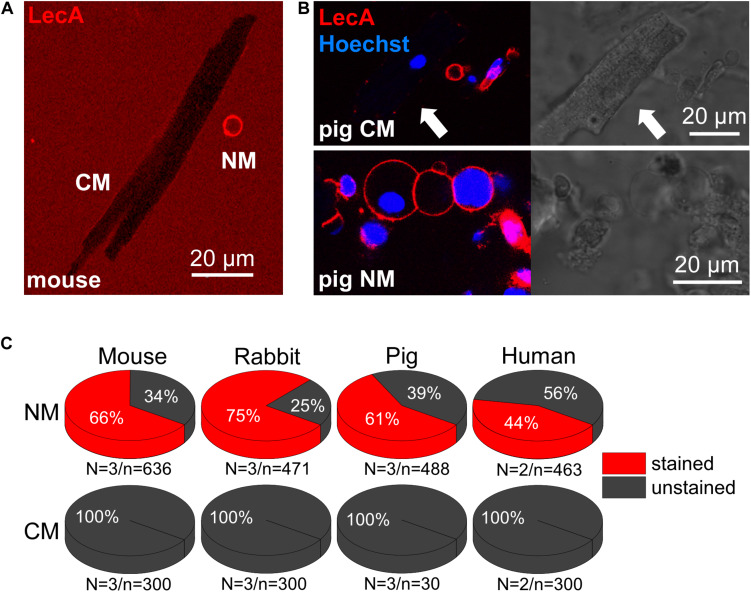
LecA binding to cardiac myocytes vs. non-myocytes. **(A,B)** Confocal images of murine **(A)** and porcine **(B)** cardiac cells (LecA: red; nuclei: blue). In contrast to non-myocytes (NM), LecA does not bind to the sarcolemma of cardiomyocytes (CM). **(C)** Quantification of LecA binding to NM (top) and CM (bottom) from mouse, rabbit, pig and human. N/n-numbers indicated below pie charts.

In freshly isolated cardiac cell suspensions from mouse, rabbit, pig and human, no LecA binding to cardiomyocytes was observed (0%, *n* = 30 for pig and 300 for the other species, Figures 6A–C). In contrast, non-myocytes exhibited intense membrane staining (66, 75, 61, and 44%; *n* > 450 for each group; Figure 6C). This differential binding is illustrated in Figures 6A,B; note: T-tubules are visible due to diffusion of labeled LecA into their lumen.

## Discussion

The present study shows that LecA binds to non-myocytes (but not to cardiomyocytes), inducing changes in surface membrane topology, and enhancing the stretch-sensitivity of TREK-1 but not of Piezo1.

### LecA Binding Dynamics and Effects on Membrane Topology

LecA binds to the plasma membrane of HEK cells within seconds. In confocal microscopy, first LecA-positive intracellular structures, compatible with endosomes, are detected 15 min after the start of LecA exposure. This time period determined the window for subsequent electrophysiological investigations.

LecA forms clusters at the cell surface. This binding pattern may be explained by the heterogeneous distribution of Gb_3_ within specific plasma membrane domains (Kovbasnjuk et al., 2001; Nutikka and Lingwood, 2003).

LecA becomes enriched over time at the interfaces between adjacent cells. This confirms a recently published observation that LecA crosslinks protocells to form prototissues (Villringer et al., 2018; Omidvar and Römer, 2019). One LecA, crosslinking two neighboring cells, might encounter less steric hindrance than binding to up to four Gb_3_ on the flat surface of a single cell. The opposing binding pockets of LecA are approximately 7 nm apart (Schubert and Römer, 2015). This distance is assumed to be large enough for crosslinking neighboring plasma membranes at points of close cell-to-cell contact. Our EM data is compatible with this possibility (just under half of the intercellular gaps are narrow enough to be bridged by LecA), and suggestive of the presence of additional mechanism(s) in regions where LecA accumulation is present in spite of inter-cellular gaps exceeding LecA size; these remain to be assessed.

Plasma membrane invaginations, colocalizing with LecA staining in our CLEM data, are highly curved membrane domains and, in places, resemble structures such as either caveolae or toxin-induced membrane tubules (Römer et al., 2007). As the voxel size of the fluorescent channel is more than 6 orders of magnitude larger than that of ET, we cannot unequivocally relate the fluorescent signal to specific ultrastructures. Beyond the in-plane resolution differences, one must bear in mind that the fluorescent signal can be generated in planes below or above the ET slices shown. That said, all membrane invaginations observed in ET occurred in membrane areas rich in LecA signal. This supports the ability of LecA (without the need for presence of LecA-producing bacteria) to trigger membrane deformations. Since such invaginations are also observed on synthetic GUV membranes, we conclude that they are not confined to cells. HEK cells endogenously express only low levels of caveolin1 (Jung et al., 2004; Pérez-Verdaguer et al., 2016) and hence lack caveolae (Figure 2A), additionally confirming the concept that LecA generates the observed membrane invaginations.

Taken together, in confocal microscopy, we observed two main LecA binding patterns: first, there are clusters (patchy pattern), possibly accounting for local negative membrane curvature (in accordance with EM data). Second, there is accumulation of LecA at cell-cell contact sites, possibly leading to cell crosslinking.

### LecA Effect on SAC

Patch-clamp recordings were performed from 2 min until 15 min after the start of LecA exposure. Electrical and mechanical stimulation protocols were applied to pre-strained membrane patches (in cell-attached configuration, membrane tension is ∼30–40% of the membrane lytic tension; Sachs, 2010), while electrical recordings were also conducted on membranes with a less disturbed mechanical environment (whole-cell configuration).

Neither AA, a well-known inducer of negative membrane curvature, nor LecA, which also causes membrane invagination, activates Piezo1. Piezo1 itself induces negative curvature in membranes, because of its curved transmembrane region (Murthy et al., 2017; Saotome et al., 2018). Stretch-activation of Piezo1 has been proposed to be due to flattening of the Piezo-related local concave dome structure (Haselwandter and MacKinnon, 2018). Thus, compounds that stabilize or enhance negative membrane curvature may prevent Piezo1 activation. Of course, membrane domains exhibiting Gb_3_, to which LecA binds, may not co-localize with domains exhibiting Piezo1.

In contrast, TREK-1 currents are increased by LecA and AA, both in response to mechanical and electrical stimulation. In cell-attached configuration, LecA effects are highly variable. This may be explained by the irregular (patchy) binding pattern of LecA to α-galactose residues. This may have given rise to substantial differences in LecA presence in any given patch clamp investigation.

The effect of LecA is modest in comparison to the activity induced by AA. This may have several explanations. Firstly, the focal effects of LecA, promoting rapid endocytosis of highly curved membrane domains, might start happening before detection with confocal microscopy. This may lead to an underestimation of its effect on the channel in case a fraction of TREK-1 is located in excised invaginations and, hence, no longer detectable in the plasma membrane-dominated electrophysiological read-outs. Secondly, the membrane curvature induced by LecA (remotely via α-galactose residues) might be less efficient in activating SAC compared to the curvature induced by changes in lipid packing generated by AA. LecA was applied at a concentration 20 times lower than AA: 500 nM for LecA (above what is required to trigger host cell signaling; see section Lectins) vs. 10 μM for AA (most commonly used concentration in publications). However, we suggest that the concentration may not be a limiting factor in LecA membrane binding as free LecA is still present in the bath solution (high fluorescence level compared to pre-LecA application) even after 30 min.

The mechanism by which LecA modifies TREK-1 activity remains an open question. The most likely scenario is mechano-activation *via* the highly curved membrane domains generated by LecA. Their presence and dimensions, with curvature radii in the range of 100 nm (which matter for SAC gating; Bavi et al., 2016) have been confirmed by our ET findings. Even though membrane topology is altered by LecA, other mechanisms could also play a role. LecA may bind directly to the channel protein or trigger secondary signaling cascades, sensitizing TREK-1. The increased response to voltage stimulation by a change in membrane mechanics is in agreement with data published for AA (Kim and Duff, 1990; Kirber et al., 1992; Fink et al., 1998), and the mechanisms underlying these matching responses to LecA and AA remain to be assessed.

To sum up, we reproduced TREK-1 activation by AA upon electrical stimulation. We also show that TREK-1 activity is increased by LecA exposure, both upon mechanical and electrical stimulation, while no effect of AA or LecA on Piezo1 was observed.

### LecA Effect on Patch Stability

Patch stability quantification reveals that LecA protects the plasma membrane, or the seal, from failure during high levels of suction. In the presence of LecA in the cell-attached configuration, recordings of Piezo1 and TREK-1 could be conducted at higher-amplitudes of negative pressure. Possible mechanisms explaining the increased stability of the patch in the presence of LecA may be stiffening of the membrane, coating of the membrane with LecA-crosslinks, or improved adhesion between glass pipette and cell membrane.

### Cell Type Selectivity of LecA Binding

Cell-preferential binding of LecA is of interest for assessment of heterocellular organs, where cell composition may vary between regions, or change as a consequence of remodeling. We observed that LecA does not bind to cardiomyocytes of four mammalian species (mouse, rabbit, pig, and human), but to non-myocytes. It may be explained by the fact that the glycocalyx composition varies between cell types (Tsimbouri et al., 2014).

LecA, or similar compounds, could provide interesting tools for cell-selective targeting in heterocellular tissue. For example, one may be able to optimize protocols where separation between cardiac myocytes and non-myocytes has been challenging so far, such as in mechano-biology research. Furthermore, being endocytosed, LecA may operate as a cell type selective transfection reagent *in vivo*.

From a pathophysiological point of view, sensitization of TREK-1 by LecA might be a starting point for novel therapeutic targeting of atrial fibrillation or heart failure – two pathologies where TREK-1 has been reported to be suppressed (Lugenbiel et al., 2017). Furthermore, TREK-1 inhibits proliferation of neuronal stem cells, astrocytes, osteoblasts, Chinese hamster ovary cells, and neonatal cardiomyocytes (Hughes et al., 2006; Xi et al., 2011; Wang et al., 2012; Yang et al., 2014; Zhang et al., 2016), but increases the proliferation of prostate cancer cell lines (Voloshyna et al., 2008). A compound that enhances TREK-1 opening might help controlling pathological tissue remodeling associated with non-myocyte proliferation in the heart. Finally, non-myocytes can be electrically coupled to cardiac myocytes (Quinn et al., 2016; Hulsmans et al., 2017). If LecA favors TREK-1 opening in cardiac non-myocytes, these cells will be more polarized, which may affect excitability of electrotonically connected cardiac myocytes.

### Limitations

LecA is a virulence factor of *P. aeruginosa* that has been shown to have cytotoxic effects in epithelial cells (Bajolet-Laudinat et al., 1994; Chemani et al., 2009), so its toxicity in cardiac non-myocytes will have to be explored. This will also determine whether the time-window, restricted in this study to ∼15 min from exposure to LecA, may be extended, which would be helpful for many experimental designs. For electrophysiological translation to the heterocellular tissue, a compound with prolonged binding to the surface membrane and reduced membrane bending effect is needed. This said, compared to traditional SAC modulators, such as lipids that insert into one leaf of the bilayer to induce curvature, LecA offers the advantage of not directly altering membrane lipid composition, limiting potential side effects.

### Outlook

In a further study, the localization of the channels (especially TREK-1) relatively to LecA and curved membrane domains will be performed. Membrane invaginations associated to LecA might colocalize with the channels. In this case, local curvature would explain the effect on the channel gating. In the opposite case, if there is no colocalization, this would suggest that an increase in the global in-plane membrane tension sensitizes the channels. Long-term effects of LecA will address whether the endocytosis observed leads to a decrease of the number of TREK-1 channels at the plasma membrane.

## Conclusion

This study demonstrates that LecA binds to non-myocytes, but not to cardiomyocytes, and changes plasma membrane topology in HEK cells. LecA increases the activity of human TREK-1 in HEK cells, in response to both mechanical and electrical stimulation, but not of human Piezo1. The here identified differential SAC modulator is potentially capable of acting in a cell type-dependent manner. It offers promise as a potential experimental tool to explore mechanotransduction in heterocellular tissues, including comparative assessments of normal and physiologically remodeled cardiac tissue.

## Data Availability Statement

The datasets generated for this study are available on request to the corresponding author.

## Ethics Statement

The studies involving human participants were reviewed and approved by Ethics Commission of the University of Freiburg, reference: 393/16 and 214/18. The patients/participants provided their written informed consent to participate in this study. The animal study was reviewed and approved by Local authorities in Baden Württemberg (X-16/10R).

## Author Contributions

PK, RP, UR, WR, JM, and ED contributed to the conception and design of the study. AB supplied the lectins and supported their experimental application. ED performed and analyzed the experiments. LS and RO performed and analyzed the GUV experiments. ER-Z acquired and analyzed the EM images. ED drafted the manuscript. All authors contributed to manuscript revision and approved the submitted version.

## Conflict of Interest

The authors declare that the research was conducted in the absence of any commercial or financial relationships that could be construed as a potential conflict of interest.

## Supplementary Material

FIGURE S1 Characterization of background activity and SAC currents in Piezo1 and TREK-1 transfected HEK cells. Patch-clamp measurements in cell-attached configuration (holding potential −80 mV for Piezo1 and 0 mV for TREK-1. **(A,C)** Representative patch-clamp recordings of non-transfected cells (top), and Piezo1 **(A)** or TREK-1 **(C)** transfected cells (middle); pressure pulses applied are 0 and −70 mmHg (bottom). **(B)** Quantification of endogenous activity at −80 mV (squares; *n* = 8 at 0 mmHg; *n* = 6 at −60 mmHg) vs. Piezo1-transfected cells (circles; *n* = 38 at 0 mmHg; *n* = 25 at −60 mmHg. **(D)** Quantification of endogenous activity at 0 mV (squares; *n* = 16 at 0 mmHg; *n* = 5 at −60 mmHg) vs. TREK-1-transfected cells (circles; *n* = 32 at 0 mmHg; *n* = 13 at −60 mmHg). Lower n-numbers at higher suction levels are caused by patches that did not withstand large pressure pulses.

FIGURE S2 Piezo1 activity in presence or absence of LecA in HEK cells. Patch-clamp measurements in cell-attached configuration (holding potential −80 mV; **A–C**) and whole-cell configuration (holding pressure 0 mmHg; **D,E**). **(A)** Analyzed parameters (applies also to TREK-1), obtained for one pulse of pressure (vertical dashed lines). From the recorded trace (black) the peak, near steady-state (green) and average (blue) current amplitudes are deduced. The exponential fit (red) yields the time constant of current inactivation. **(B–D)** Quantification of the activity induced by LecA (2 min; *n* = 37) vs. tmControl (*n* = 38). **(E)** Quantification of the activity induced by LecA over 200 s of LecA exposure (*n* = 7 for all data). **(F)** Single data points at 80 s are shown. Significance was assessed by the Mann–Whitney-test.

FIGURE S3 TREK-1 activity in the absence or presence of LecA in HEK cells. Patch-clamp measurements in cell-attached configuration (holding potential 0 mV). **(A–C)** Quantification of the activity induced by LecA (2 min; *n* = 46; in red) vs. tmControl (*n* = 43; in black). **p* ≤ 0.05.

FIGURE S4 TREK-1 activity in the absence of presence of arachidonic acid (AA) in HEK cells. Patch-clamp measurements in whole-cell configuration (holding pressure 0 mmHg); control: black, AA (10 μM) in the extracellular bath solution: red, wash-out: green. **(A)** Representative recording; Top: 80 s after onset of AA perfusion; pre-drug control at 10 s before onset of AA perfusion; After 90 s of wash-out; Middle: Difference current (blue); pre-drug control activity subtracted from AA activity; Bottom: voltage ramp applied from −80 to +40 mV. **(B)** Quantification of the TREK-1 activation normalized to the pre-drug control, for AA (*n* = 9 at 0 s; *n* = 5 at 80 s) vs. tmControl (*n* = 4 at 0 and 80 s), 0 s corresponds to the onset of AA perfusion. **(C)** Single data points at 55 s are shown. Significance was assessed by the Mann–Whitney-test. **(D)** Quantification of the reversibility of the AA effect. The current during wash-out (I_wash–out_) is normalized to the peak current reached during AA perfusion (I_AA peak_), for wash-out effect at 5 s (*n* = 8) vs. 90 s (*n* = 3), 0 s corresponds to the onset of wash-out perfusion. **(E)** Single data points at 5 and 90 s are shown. Significance was assessed by the Mann–Whitney-test. **p* ≤ 0.05.

FIGURE S5 Piezo1 activity in the absence or presence of arachidonic acid (AA) in HEK cells. Patch-clamp measurements in whole-cell configuration (holding pressure 0 mmHg); AA (10 μM) in the bath solution: red, tmControl: black. **(A)** Quantification of Piezo1 activity during AA exposure (*n* = 6 at 0 s; *n* = 3 at 80 s) vs. tmControl (*n* = 4 at 0 s; *n* = 3 at 80 s). **(B)** Single data points at 80 s are shown. Significance was assessed by the Mann–Whitney-test.
