# Supplementary figures and images for: The Lectin LecA Sensitizes the Human Stretch-Activated Channel TREK-1 but Not Piezo1 and Binds Selectively to Cardiac Non-myocytes
Source: Front Physiol. 2020 May 15;11:457. doi: 10.3389/fphys.2020.00457 (PMC7243936; doi:10.3389/fphys.2020.00457)

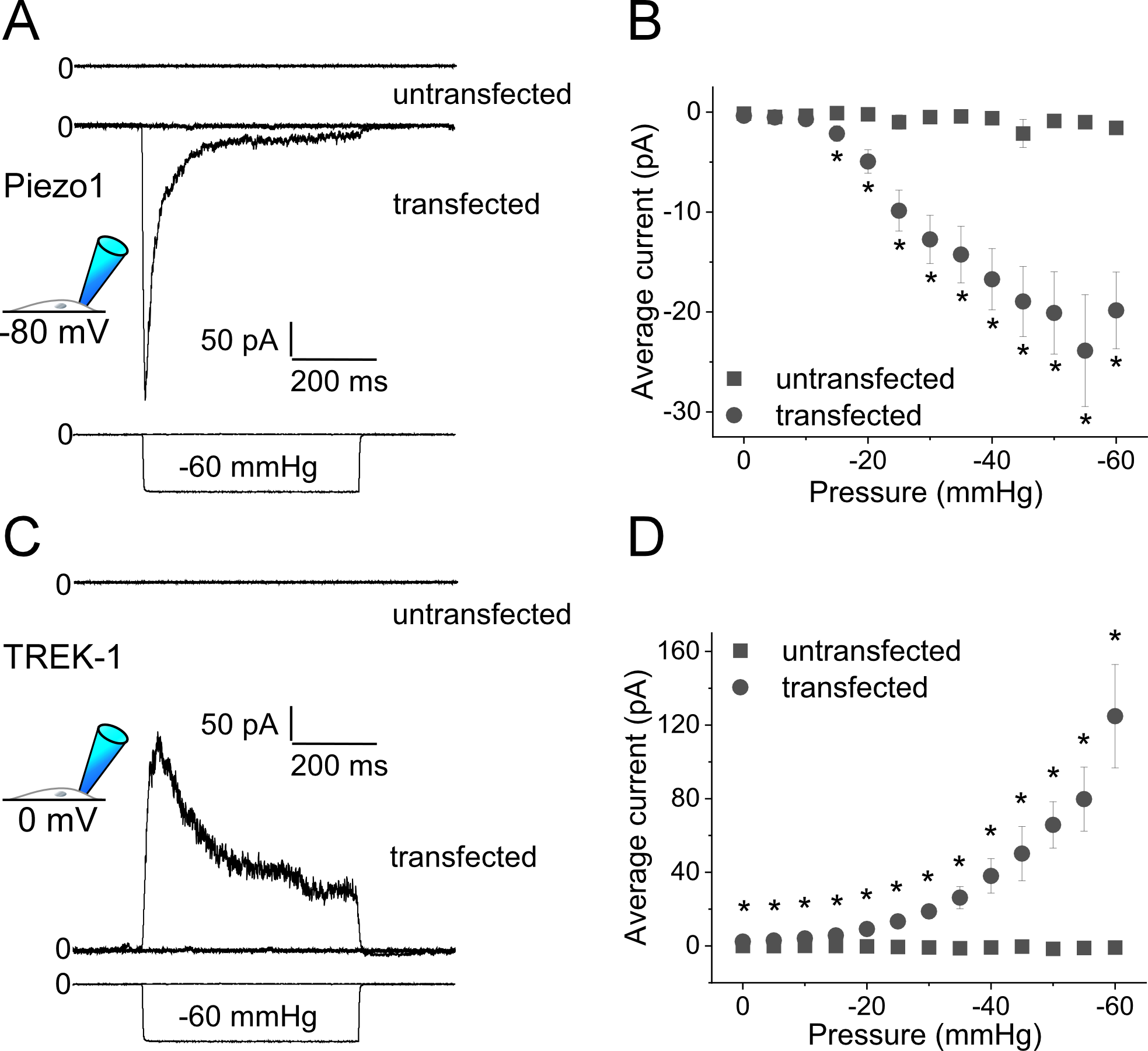

Supplement: FIGURE S1 — Characterization of background activity and SAC currents in Piezo1 and TREK-1 transfected HEK cells. Patch-clamp measurements in cell-attached configuration (holding potential −80 mV for Piezo1 and 0 mV for TREK-1. (A,C) Representative patch-clamp recordings of non-transfected cells (top), and Piezo1 (A) or TREK-1 (C) transfected cells (middle); pressure pulses applied are 0 and −70 mmHg (bottom). (B) Quantification of endogenous activity at −80 mV (squares; n = 8 at 0 mmHg; n = 6 at −60 mmHg) vs. Piezo1-transfected cells (circles; n = 38 at 0 mmHg; n = 25 at −60 mmHg. (D) Quantification of endogenous activity at 0 mV (squares; n = 16 at 0 mmHg; n = 5 at −60 mmHg) vs. TREK-1-transfected cells (circles; n = 32 at 0 mmHg; n = 13 at −60 mmHg). Lower n-numbers at higher suction levels are caused by patches that did not withstand large pressure pulses. [file Image_1.TIF]

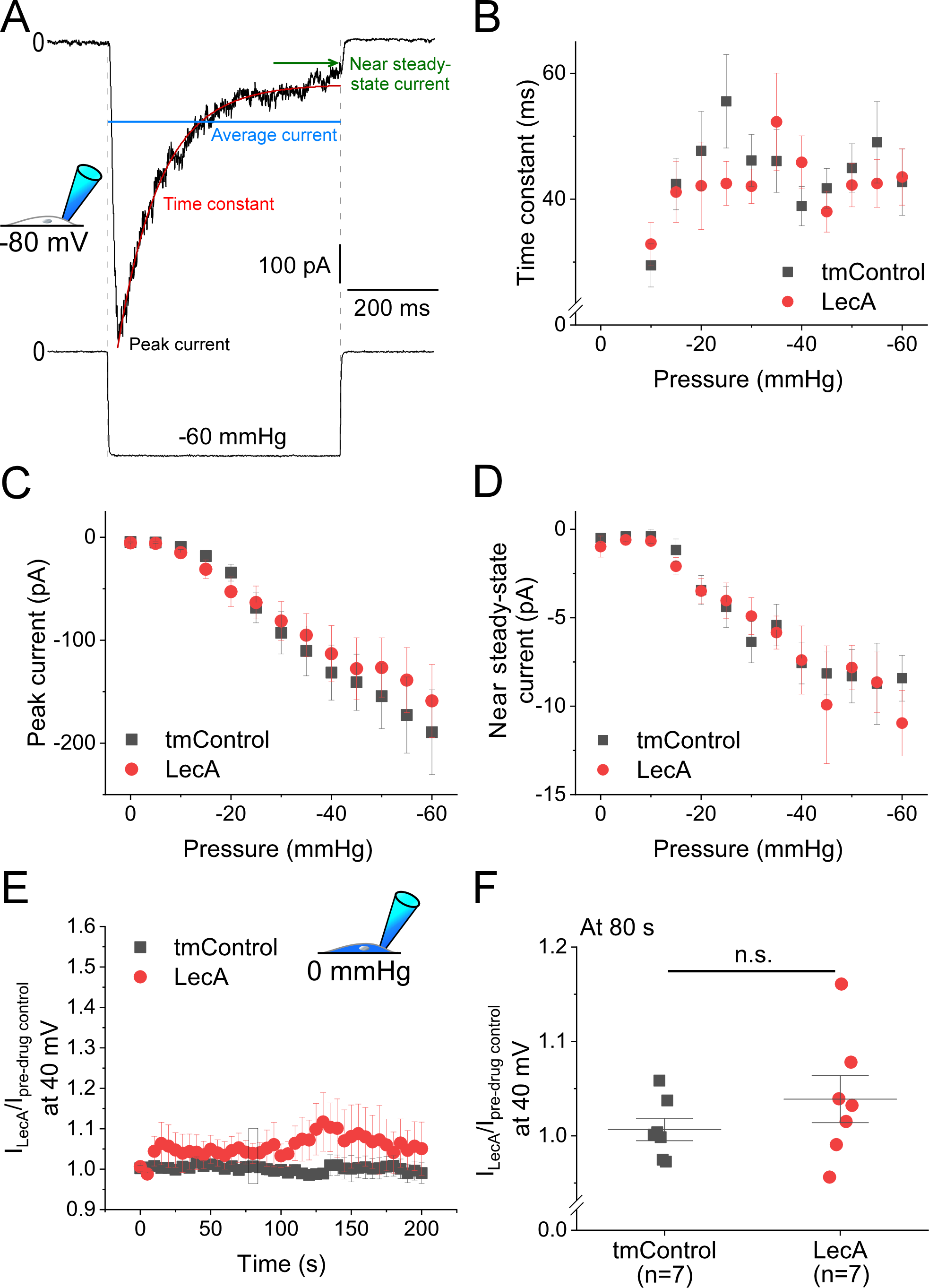

Supplement: FIGURE S2 — Piezo1 activity in presence or absence of LecA in HEK cells. Patch-clamp measurements in cell-attached configuration (holding potential −80 mV; A–C) and whole-cell configuration (holding pressure 0 mmHg; D,E). (A) Analyzed parameters (applies also to TREK-1), obtained for one pulse of pressure (vertical dashed lines). From the recorded trace (black) the peak, near steady-state (green) and average (blue) current amplitudes are deduced. The exponential fit (red) yields the time constant of current inactivation. (B–D) Quantification of the activity induced by LecA (2 min; n = 37) vs. tmControl (n = 38). (E) Quantification of the activity induced by LecA over 200 s of LecA exposure (n = 7 for all data). (F) Single data points at 80 s are shown. Significance was assessed by the Mann–Whitney-test. [file Image_2.TIF]

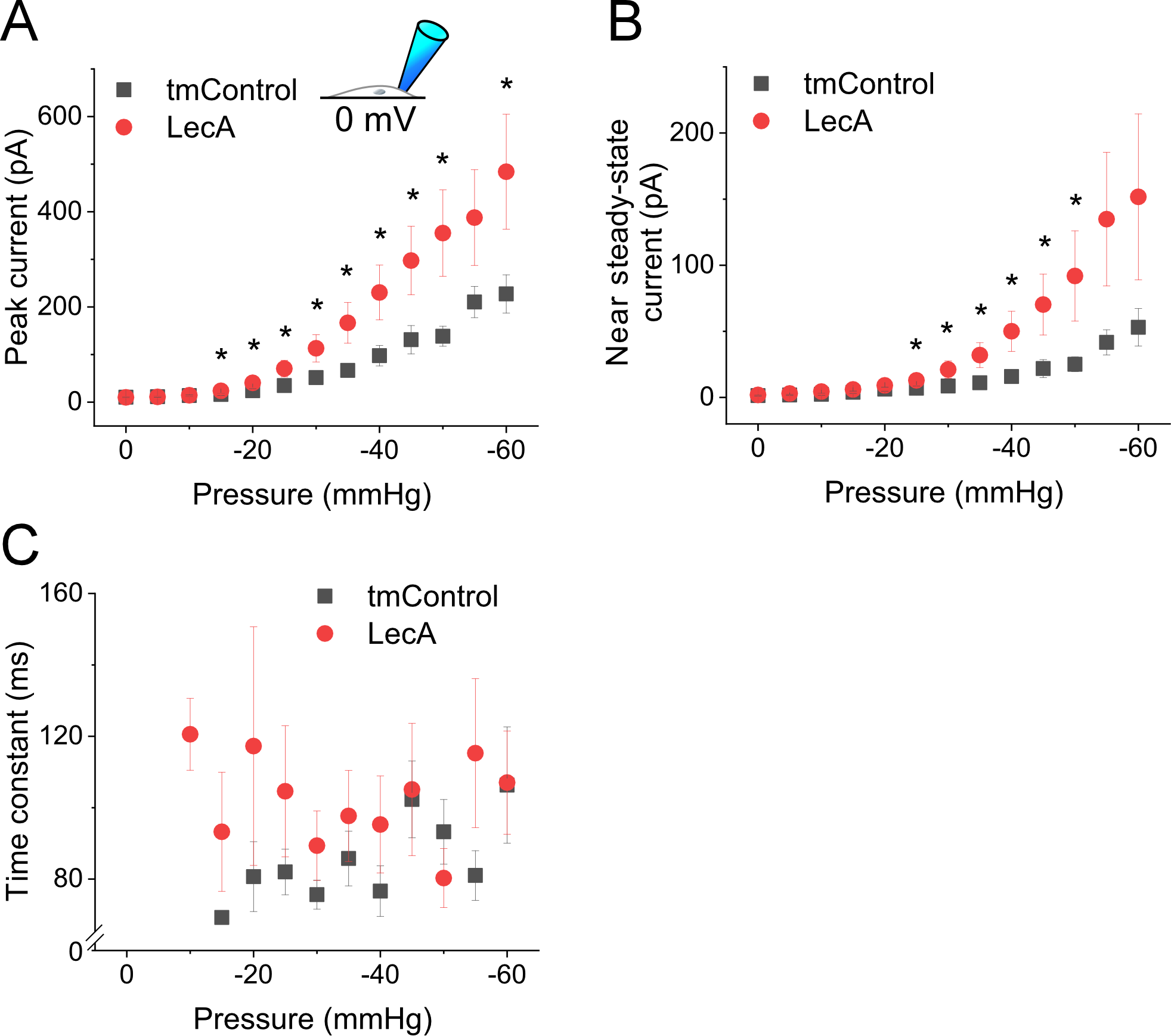

Supplement: FIGURE S3 — TREK-1 activity in the absence or presence of LecA in HEK cells. Patch-clamp measurements in cell-attached configuration (holding potential 0 mV). (A–C) Quantification of the activity induced by LecA (2 min; n = 46; in red) vs. tmControl (n = 43; in black). *p ≤ 0.05. [file Image_3.TIF]

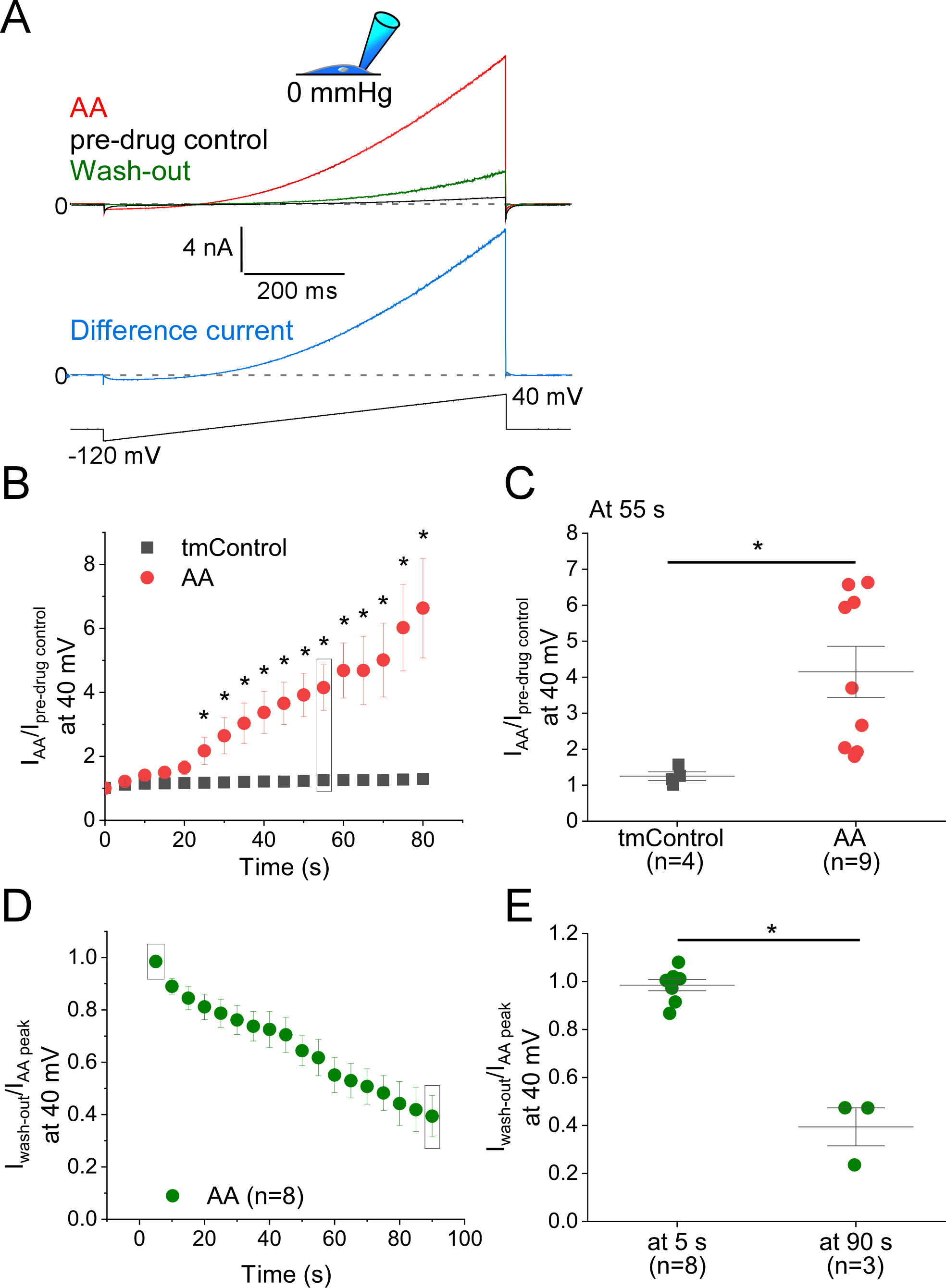

Supplement: FIGURE S4 — TREK-1 activity in the absence of presence of arachidonic acid (AA) in HEK cells. Patch-clamp measurements in whole-cell configuration (holding pressure 0 mmHg); control: black, AA (10 μM) in the extracellular bath solution: red, wash-out: green. (A) Representative recording; Top: 80 s after onset of AA perfusion; pre-drug control at 10 s before onset of AA perfusion; After 90 s of wash-out; Middle: Difference current (blue); pre-drug control activity subtracted from AA activity; Bottom: voltage ramp applied from −80 to +40 mV. (B) Quantification of the TREK-1 activation normalized to the pre-drug control, for AA (n = 9 at 0 s; n = 5 at 80 s) vs. tmControl (n = 4 at 0 and 80 s), 0 s corresponds to the onset of AA perfusion. (C) Single data points at 55 s are shown. Significance was assessed by the Mann–Whitney-test. (D) Quantification of the reversibility of the AA effect. The current during wash-out (Iwash–out) is normalized to the peak current reached during AA perfusion (IAA peak), for wash-out effect at 5 s (n = 8) vs. 90 s (n = 3), 0 s corresponds to the onset of wash-out perfusion. (E) Single data points at 5 and 90 s are shown. Significance was assessed by the Mann–Whitney-test. *p ≤ 0.05. [file Image_4.TIF]

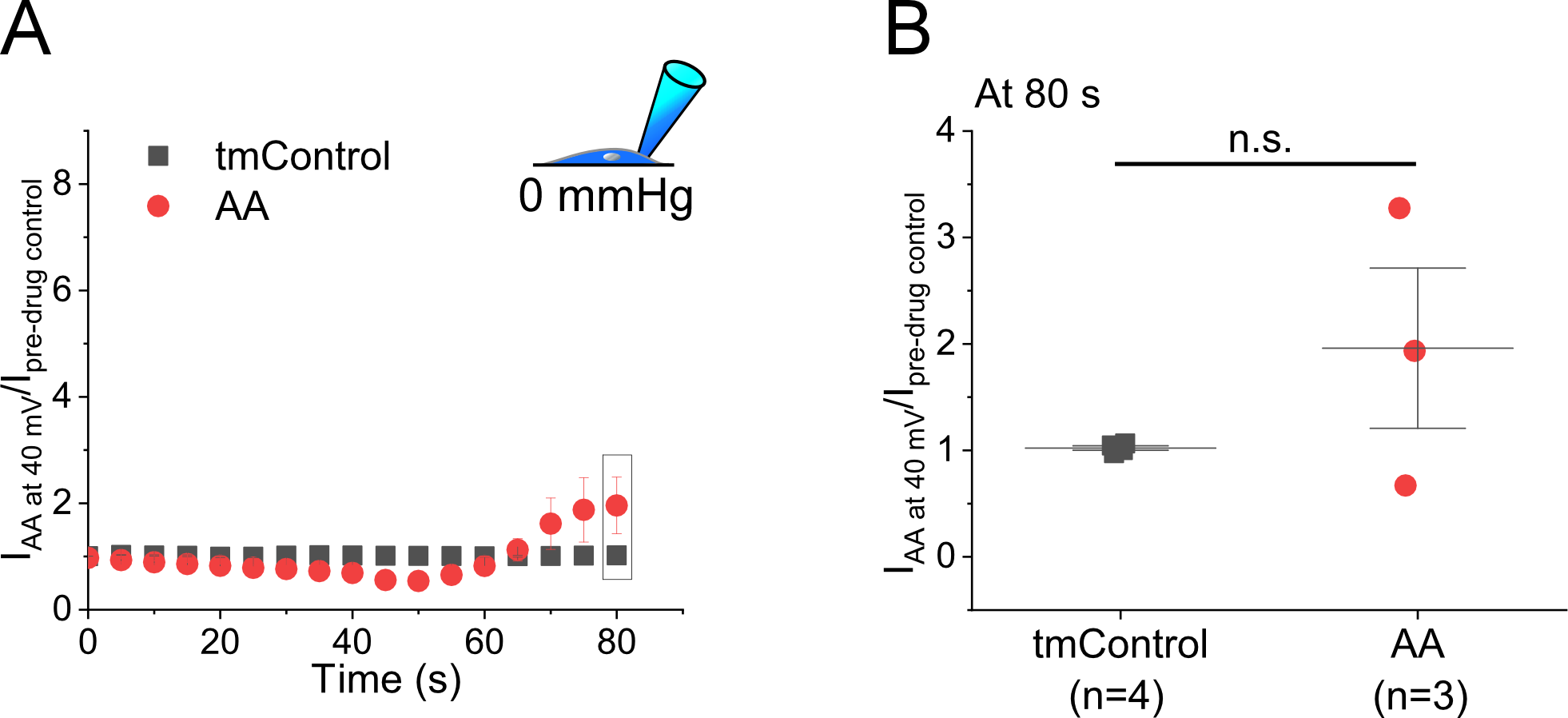

Supplement: FIGURE S5 — Piezo1 activity in the absence or presence of arachidonic acid (AA) in HEK cells. Patch-clamp measurements in whole-cell configuration (holding pressure 0 mmHg); AA (10 μM) in the bath solution: red, tmControl: black. (A) Quantification of Piezo1 activity during AA exposure (n = 6 at 0 s; n = 3 at 80 s) vs. tmControl (n = 4 at 0 s; n = 3 at 80 s). (B) Single data points at 80 s are shown. Significance was assessed by the Mann–Whitney-test. [file Image_5.TIF]
